# The Opportunity of Proteomics to Advance the Understanding of Intra- and Extracellular Regulation of Malignant Hematopoiesis

**DOI:** 10.3389/fcell.2022.824098

**Published:** 2022-03-08

**Authors:** Maria Jassinskaja, Jenny Hansson

**Affiliations:** ^1^ Lund Stem Cell Center, Division of Molecular Hematology, Lund University, Lund, Sweden; ^2^ York Biomedical Research Institute, Department of Biology, University of York, York, United Kingdom

**Keywords:** leukemia, hematopoiesis, developmental hematopoiesis, proteomics, post-transcriptional regulation

## Abstract

Fetal and adult hematopoiesis are regulated by largely distinct sets of cell-intrinsic gene regulatory networks as well as extracellular cues in their respective microenvironment. These ontogeny-specific programs drive hematopoietic stem and progenitor cells (HSPCs) in fetus and adult to divergent susceptibility to initiation and progression of hematological malignancies, such as leukemia. Elucidating how leukemogenic hits disturb the intra- and extracellular programs in HSPCs along ontogeny will provide a better understanding of the causes for age-associated differences in malignant hematopoiesis and facilitate the improvement of strategies for prevention and treatment of pediatric and adult acute leukemia. Here, we review current knowledge of the intrinsic and extrinsic programs regulating normal and malignant hematopoiesis, with a particular focus on the differences between infant and adult acute leukemia. We discuss the recent advances in mass spectrometry-based proteomics and its opportunity for resolving the interplay of cell-intrinsic and niche-associated factors in regulating malignant hematopoiesis.

## Introduction

Over the past 50 years, the overall survival rates for childhood acute lymphoblastic leukemia (cALL) have increased from a mere 10% to nearly 90% (Malard and Mohty, 2020). However, current treatment schemes involve intensive chemotherapy, which is associated with severe, sometimes life-long, side effects (Blaauwbroek et al., 2007). Even the recently approved chimeric antigen receptor (CAR)-T cell therapy is associated with considerable adverse systemic effects (Sermer and Brentjens, 2019). Additionally, the prognosis for the youngest leukemia patients (<1 year) remains dismal with an overall survival rate below 50% (Pui et al., 2002; Pieters et al., 2007). As such, there is a critical need for development of novel, targeted therapies. Attempts at developing such therapies are currently hindered by an incomplete understanding of the molecular mechanisms at play in leukemic cells and their environment during initiation and progression of the disease. With latest advancements in mass spectrometry (MS)-based proteomics, a critical opportunity for resolving how leukemogenic hits disturb the intra- and extracellular programs in HSPCs along ontogeny has arisen. In this review, we summarize current knowledge of the intrinsic and extrinsic programs regulating normal and malignant hematopoiesis, with a particular focus on the differences between infant and adult acute leukemia. Finally, we discuss how proteomics can accelerate this understanding.

## Intra- and Extracellular Regulation of Hematopoiesis

### Fetal and Adult-specific Regulators of HSPC Function and Lymphomyeloid Differentiation

**FIGURE 1:**
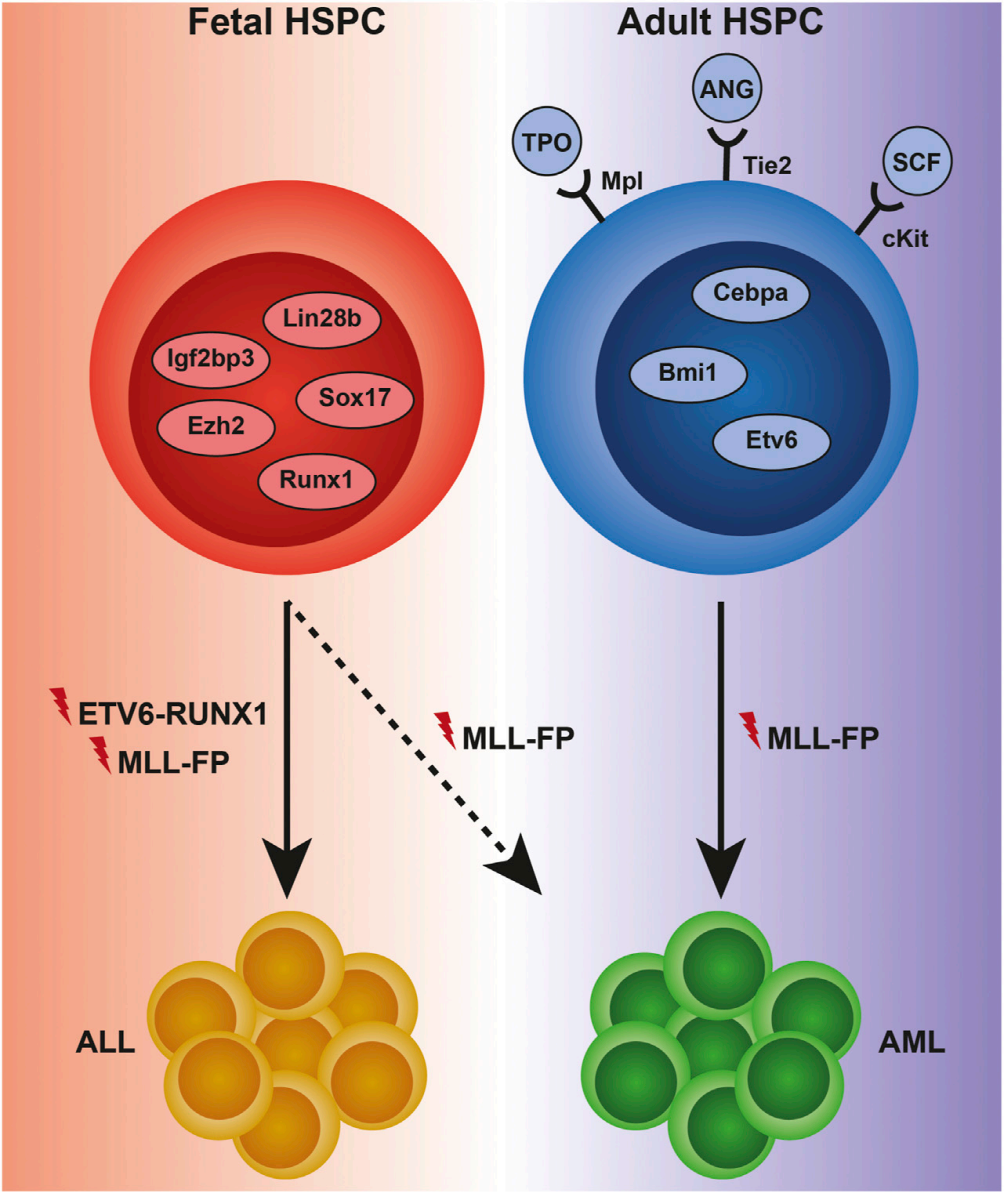
Examples of intra- and extracellular regulators of normal and malignant hematopoiesis across ontogeny. HSPCs in the fetus are dependent on proteins such as Lin28b, Sox17, Igf2bp3, Ezh2, and Runx1 to maintain a high self-renewal capacity, as well as for differentiation towards mature cells, including fetal-specific immune cell subsets. Adult HSPCs display a strict requirement for quiescence, which is maintained by proteins such as Cebpa, Bmi1, and Etv6 intracellularly, and thrombopoietin (TPO), angiopoietin (ANG), and stem cell factor (SCF) extracellularly. The changing composition of the HSPC proteome to a large extent controls the susceptibility to initiation and progression of leukemia. Genetic translocations leading to the expression of the ETV6-RUNX1-and MLL-fusion proteins (FPs) almost invariably result in acute lymphoblastic leukemia (ALL) when occurring during fetal development, although rare cases of MLL-FP-driven infant AML do occur. In contrast, MLL-fusions in adult HSPCs almost invariably cause acute myeloid leukemia (AML).

Fetal and adult hematopoiesis are regulated by largely distinct sets of gene regulatory networks (GRNs) and extracellular cues which enforce and maintain the key features of hematopoiesis at different developmental stages: in fetus—expansion, and in adult—homeostasis (Figure 1). Two of the most potent drivers of a fetal-like state of hematopoiesis are Lin28b and Igf2bp3 (Yuan et al., 2012; Wang et al., 2019), and ectopic expression of either protein is sufficient to revert adult hematopoietic stem and progenitor cells (HSPCs) back to a fetal-like state (Yuan et al., 2012; Rowe et al., 2016; Wang et al., 2019). The high self-renewal activity of fetal hematopoietic stem cells (HSCs) is additionally dependent on the genes Sox17 and Ezh2, both of which are largely dispensable for adult HSC function (Kim et al., 2007; Mochizuki-Kashio et al., 2011). Fetal hematopoiesis towards all lineages additionally requires intact expression of Runx1 (Okuda et al., 1996), while adult Runx1-deficient HSCs exhibit differentiation defects but are viable (Ichikawa et al., 2004). Instead, the presence of, for example, Gfi1, ETS translocation variant 6 (ETV6), Bmi1 and CCAAT/enhancer binding protein (CEBP) α, is strictly required for survival and self-renewal of adult, but not fetal, HSCs (Park et al., 2003; Hock et al., 2004a, 2004b; Ye et al., 2013).

As fetal and adult HSPCs reside in distinct niches (the fetal liver [FL] and the adult bone marrow [ABM], respectively), it is perhaps unsurprising that the cells exhibit ontogeny-specific dependencies on extracellular factors for survival and function. Examples include thrombopoietin (TPO), angiopoietin and stem cell factor (SCF) which are required for ABM HSC quiescence and survival, while deficiency of either factor or its receptor (Mpl, Tie2 and cKit, respectively) has less impact on FL hematopoiesis (Puri and Bernstein, 2003; Qian et al., 2007; Thorén et al., 2008).

Differentiation and mature cell production are governed by a complex interplay of transcription factors (TFs) and extracellular stimuli in the form of cytokines and receptor-ligand interactions with niche cells. In the adult, generation of common lymphoid progenitors (CLPs), the common ancestor of B, T and natural killer (NK) cells (Kondo et al., 1997), requires signaling through cKit and fms-like tyrosine kinase 3 (Flt3) via their respective ligands (Sitnicka et al., 2002; Waskow et al., 2002). B, T and NK cell development is governed by three largely distinct networks of TFs, which include PU.1, Ikaros (Ikzf1), Gfi1, Tcf3, Foxo1, Ebf1, and Pax5 for B cells (Somasundaram et al., 2015), Nfil3, Id2, Ets1, Eomes, and T-bet for NK cells (Kee et al., 2020), and Tcf1, Gata3, and Bcl11b for T cells (Yui and Rothenberg, 2014). Notably, the generation of innate-like B1 B cells during fetal life does not require PU.1 (Ye et al., 2005), but is dependent on Lin28b-mediated expression of Arid3a (Zhou et al., 2015). Similarly, Bcl11b is dispensable for the generation of fetal-specific subsets of γδT cells (Li et al., 2010).

While myeloid potential is nearly completely lost at the CLP stage of lymphopoiesis *in vivo*, myeloid programs have been shown to be partially reactivated during *ex vivo* culture of adult CLPs under lymphomyeloid differentiation conditions (Richie Ehrlich et al., 2011). We have recently shown that this is not the case for fetal CLPs, which retain lymphoid restriction in the presence of myelopoiesis-promoting cytokines in culture, and, accordingly, express lower levels of proteins associated with myelopoiesis than their adult counterparts (Jassinskaja et al., 2021).

Initiation and propagation of myeloid programs in multipotent progenitors (MPPs) is governed mainly by the TFs PU.1, CEBPα and ε, Gfi1 and Irf8 (Rosenbauer and Tenen, 2007). Irf8 is a highly important regulator of lineage choices between neutrophils, monocytes and dendritic cells (DCs), and represents an example of a TF driving lineage commitment in a dose-dependent manner; lack of this TF promotes neutrophil generation whereas low and high levels drive differentiation towards inflammatory Ly6C^+^ monocytes and DCs, respectively (Murakami et al., 2021). In line with this, fetal granulocyte-monocyte progenitors (GMPs), which express low levels of Irf8 protein (Jassinskaja et al., 2021), have limited potential to produce fully mature inflammatory monocytes compared to adult GMPs. Intriguingly, a recent study comparing human FL and fetal bone marrow hematopoiesis revealed that myeloid potential is remarkably low in the former relative to the latter (Popescu et al., 2019; Jardine et al., 2021), further highlighting the differential lineage bias of HSPCs derived from different stages of ontogeny. Extracellularly, adult myelopoiesis is strongly stimulated by cytokines such as granulocyte-colony stimulating factor (G-CSF), macrophage (M)-CSF and interleukin (IL)-6 (Nakamura et al., 2004; Rosenbauer and Tenen, 2007).

As outlined above, known differences exist in the dependency of fetal and adult HSPCs on TFs considered as fundamental components of GRNs regulating lymphomyeloid differentiation, as well as on extracellular stimuli. Further characterization of the molecular programs active in fetal HSPCs is necessary in order to fully understand ontogeny-specific factors governing mature cell output at different developmental stages.

### Post-Transcriptional Regulation in Fetal and Adult Hematopoiesis

Several bodies of recent work, including studies performed in our lab, have shown that cellular processes such as metabolism, response to inflammation and even malignant transformation are regulated post-transcriptionally in HSPCs (Cabezas-Wallscheid et al., 2014; Haas et al., 2015; Jassinskaja et al., 2017, 2021; Raffel et al., 2020). Furthermore, adult HSCs, which are hallmarked by a low protein synthesis rate (Signer et al., 2014), have recently been shown to have a significantly higher rate of transcription compared to more downstream hematopoietic progenitor cells (HPCs) (Mansell et al., 2021). This suggests that while mRNA-based analysis can provide an accurate picture of the cells’ potential, it is an inadequate predictor of the actual cellular phenotype in quiescent HSCs. In line with this, the correlation between protein and mRNA expression is particularly poor in HSCs, although that observed in MPPs is only slightly higher (Zaro et al., 2020). Comparison of protein data generated in our lab of fetal and adult lymphomyeloid multipotent progenitors (LMPPs) (Jassinskaja et al., 2021) to a recently published RNAseq dataset of the same populations (Symeonidou et al., 2021) further highlights that correlation between proteome and transcriptome is mediocre at best even in progenitors downstream of HSCs (*r* = 0.4216). In addition, a very recent study even highlights a non-negligible role for protein-level diversity by the individual proteoforms in hematopoiesis (Melani et al., 2022). Maintaining a diverse transcriptome may facilitate lineage plasticity in stem cells and MPPs, allowing for rapid translation of the appropriate transcripts and subsequent lineage commitment and mature blood cell production in response to the need of the organism, as recently proposed by Mansell et al. This theory is corroborated by reports showing that upon inflammation-induced emergency megakaryopoiesis, proteins associated with a megakaryocytic cell fate are upregulated in HSCs, while mRNA levels of the same genes are unchanged (Haas et al., 2015). Accurate characterization of the inflammatory response in HSPCs is of particular importance, as inflammation has been shown to play a profound role in the regulation of fetal as well as adult hematopoiesis (Demerdash et al., 2021). Differential expression of Type I Interferon response genes at different ontogenic stages, such as that previously reported by our lab (Jassinskaja et al., 2017), may additionally carry clinical relevance as the proliferation-promoting effect of interferons (IFNs) have been utilized in the treatment of leukemia, where administration of IFN is believed to drive dormant leukemic cells into cycle, thus sensitizing them to killing by chemotherapeutic agents (Essers et al., 2009).

## Infant and Childhood Acute Leukemia

### Mutations and Disease Subtypes

Infant (<1 year of age) and childhood (<18 years of age) acute leukemias are often initiated already *in utero* (Ford et al., 1993; Greaves et al., 2003). Several different first-hit mutations acting as drivers of infant and childhood ALL have been identified. The most prevalent mutation in infant ALL involves fusion of the mixed lineage leukemia (MLL) gene with one of its many partner genes (discussed below), while the fusion of ETV6 and RUNX1 genes is the most common driver mutation in cALL (Iacobucci and Mullighan, 2017) (Figure 1). The ETV6-RUNX1 translocation is present in approximately 1% of newborns but requires additional mutational events for disease to develop; thus, most children born with the translocation never develop overt leukemia (Mori et al., 2002). In stark contrast, MLL-translocations are highly potent oncogenes that rarely require additional mutations to produce aggressive leukemias. MLL-rearranged (MLLr) ALL has a peak incidence in children below 2 years of age, indicating that leukemic transformation is complete or nearly complete even before birth. This peak incidence is followed by a sharp drop during later childhood and a modest increase in the elderly population. The MLLr incidence rate for lymphoid and myeloid leukemias is remarkably different between young children and adults. MLLr acute myeloid leukemia (AML) is rarely observed in infants and is instead more common in adults over 60 years of age (Meyer et al., 2017). MLL-translocations are present in as much as 80% of infant ALL, and in approximately 50% of the few infant AML cases that do occur (Hilden et al., 2006; Pieters et al., 2007). Perplexingly, *de novo* MLLr acute leukemias are relatively rare in adults, where MLL-translocations are instead more commonly associated with AML arising following treatment with chemotherapeutic agents (Ezoe, 2012). Even when accounting for secondary leukemias, MLL-rearrangements are found in only 9% of all adult acute leukemia cases (Britten et al., 2019). Regardless, the presence of an MLL-translocation is a predictor of poor outcome in infant as well as in adult leukemia (Pui et al., 2002; Ezoe, 2012).

Few concrete predisposing factors have been identified in infant and childhood leukemia. Exposure to ionizing radiation is the only environmental factor that has been relatively consistently linked to an increased risk of pediatric leukemia (Schüz and Erdmann, 2016). For children born with the ETV6-RUNX1 translocation, a strong correlation has additionally been observed between delayed exposure to infection in early postnatal life and the risk of acquiring secondary mutations which subsequently lead to the development of cALL (Greaves, 2018). However, in MLLr as well as in ETV6-RUNX1-driven leukemia, the environmental or hereditary factors promoting the acquisition of the initial driver mutation *in utero*, if any such factors exist, remain elusive.

### Molecular Features of MLLr Infant and Adult Leukemia

Close to 80 fusion partners of the MLL gene have been identified to date, but only 5 of all known MLL-fusions account for 80% of MLLr leukemia cases. In infant as well as in adult MLLr leukemia, ALL1-fused gene from chromosome 4 and chromosome 9 (AF4 and AF9, respectively) represent the most common fusion partners of MLL. For other MLL fusion partners, however, distinct age-associated patterns have been observed. The fusion of MLL with the gene eleven-nineteen leukemia (ENL) is present in 18% of infant MLLr cases, but only in 8% of adult cases. This fusion protein (FP) is additionally associated with different disease phenotypes in infant and adult MLLr leukemia: the MLL-ENL fusion is found in 22% of infant, but only 12% of adult, ALL cases, and is very rarely observed in infant MLLr AML. In contrast, MLL-AF9 is frequently found in infant ALL as well as AML but is almost exclusively associated with AML in adults (Meyer et al., 2017). The reason behind the different behavior of specific MLL-FPs in infant an adult leukemia remains a matter of open investigation.

Compared to other leukemias, MLLr leukemia is hallmarked by a highly distinct gene expression profile (Armstrong et al., 2001). By driving expression of genes such as the Hox genes and Meis1, MLL-fusions confer cells with stem cell-like properties (Zeisig et al., 2004), including acquisition of self-renewal capacity in otherwise short-lived lineage-committed HPCs which can then function as potent leukemia stem cells (LSCs) (Cozzio et al., 2003; Krivtsov et al., 2006; Ugale et al., 2014, 2017). Upon expression of MLL-AF9 in GMPs, a large number of HSC-associated genes are re-activated (Krivtsov et al., 2006). More recent work has outlined two distinct sets of MLL-ENL target genes, which involve interaction of the FP with either chromatin modifier DOT1L or positive transcription elongation complex b (P-TEFb). The interaction leads to enhanced expression of leukemia-associated TFs (e.g. HoxA cluster genes, Meis1 and Mecom) and genes involved in protein translation (e.g. Myc), respectively (Garcia-Cuellar et al., 2016). In line with the induced differentiation arrest in cells transformed by MLL-fusions (Zeisig et al., 2004), the expression of genes associated with lineage commitment are downregulated in MLL-FP-expressing LSCs (Garcia-Cuellar et al., 2016).

Similarly to normal hematopoiesis, proteome level characterization of the molecular events governing MLL-FP-mediated leukemogenesis in infants and adults remains scarce. This is of particular concern considering the discrepancies between proteome and transcriptome in HSPCs outlined above (Cabezas-Wallscheid et al., 2014; Haas et al., 2015; Jassinskaja et al., 2017; Zaro et al., 2020), as well as the numerous reports showing even further decrease in mRNA-protein correlation upon malignant transformation (Yang et al., 2020). Indeed, the expression of genes associated with metabolic rewiring, a key hallmark of cancer, was recently shown to be regulated post-transcriptionally in primary human AML (Raffel et al., 2017, 2020). It is thus highly likely that much of the molecular programs driving leukemia initiation and development remains shrouded in darkness due to insufficient availability of protein-level information.

### Cell of Origin in Infant and Adult MLLr Leukemia

Despite decades of research, no consensus has yet been reached about the exact identity of the leukemia initiating cell (LIC) in MLLr leukemia. While a host of different HSPCs, ranging from HSCs to committed T cell progenitors, have been nominated as potential LICs in adult MLLr AML (Chen et al., 2008; Krivtsov et al., 2012; Ugale et al., 2014, 2017), far less is known about the cell of origin in infant MLLr leukemia (Milne, 2017). Age-specific cell characteristics have been shown to play a detrimental role in leukemias driven by the NUP98-HOXA9 (Chaudhury et al., 2018), ETV6-RUNX1 (Böiers et al., 2018), and ETO2-GLIS2 (Lopez et al., 2019) oncogenes. This has also been suggested for some MLLr leukemias, where MLLr lymphoid leukemia has only been successfully produced from murine FL cells (Chen et al., 2011), human cord blood cells (Barabé et al., 2007; Wei et al., 2008; Buechele et al., 2015) and, most recently, human FL HSPCs (Rice et al., 2021). Additionally, a recent study has shown that fetal and neonatal HSPCs are more susceptible to MLL-ENL-mediated transformation than their adult counterpart (Okeyo-Owuor et al., 2019), providing a possible explanation for the higher prevalence of MLL-rearrangements in infant relative to adult acute leukemia. Other work has demonstrated a profound role of the developmental stage of the niche in determining disease phenotype, and that the neonatal microenvironment more efficiently drives a lymphoid-like leukemia compared to the ABM (Rowe et al., 2019). Collectively, these studies highlight the importance of the developmental stage from which the LIC is derived and in which the leukemia is propagated in determining disease phenotype and progression. Further exploration of the molecular makeup of fetal and adult HSPCs as well as their microenvironment is critical in order to elucidate the factors responsible for the cells’ differential susceptibility to initiation of MLLr ALL and AML.

## Mass Spectrometry-Based Quantitative Proteomics

### Data-dependent and Data-independent Acquisition Mass Spectrometry

Mass spectrometry (MS)-based proteomics is the most powerful tool for interrogating and comparing different cell types or cellular states at the level of the main functional units of the cells—the proteins. While global characterization and relative comparison of cellular proteomes traditionally have been performed in data-dependent acquisition (DDA) mode, data-independent acquisition (DIA) has rapidly gained grounds in the past few years. In the former, only the most abundant peptide ions are selected for subsequent fragmentation, meaning that low-abundant peptides escape identification. In contrast, in DIA, fragmentation is performed on all ions that are present within a given mass over charge (m/z) range (Zhang et al., 2013), representing a significantly less biased approach for protein identification and quantification (Ludwig et al., 2018). The simplicity of implementation of DIA approaches and their utility is improving, through advancements in DIA acquisition protocols, algorithms, and software (Demichev et al., 2019; Bekker-Jensen et al., 2020), together with continuous developments in high resolution MS.

Sample preparation for MS-based protein quantification can be achieved by an array of different methods which are associated with more or less manipulation of cells, proteins or peptides. Label-free quantification (LFQ), which does not introduce any labeling reagents during sample preparation, can be implemented in DDA as well as DIA approaches (Ludwig et al., 2018). Labeling approaches include metabolic labeling by stable isotope labeling of amino acids in culture (SILAC) (Ong et al., 2002) and chemical labeling, which is most often done at the peptide level (Thompson et al., 2003; Dayon et al., 2008; Pierce et al., 2008; Boersema et al., 2009; Werner et al., 2014), of up to 18 different samples in one MS experiment (termed TMTPro) (Li et al., 2020, 2021). Initial issues with quantitative accuracy of TMT-based approaches have been solved by innovative MS methodologies (Rauniyar and Yates, 2014) and recent developments in gas phase fractionation technology (Schweppe et al., 2019). Interestingly, TMT-multiplexing was recently combined with DIA acquisition (Ctortecka et al., 2021).

### Moving Towards Proteomics at the Single HSPC Level

Unlike mRNA, the protein content of a cell cannot be amplified in the test tube. Thus, comprehensive proteomic characterization of rare cell types, such as primary HSPCs which are challenging to expand *in vitro* (Walasek et al., 2012), has historically been difficult. MS instrumentation has long possessed sufficient sensitivity for peptide detection at or close to the single-cell level. However, in order to resolve complex protein mixtures with a dynamic range spanning at least seven orders of magnitude in the case of a mammalian cellular proteome (Zubarev, 2013), considerable pre-processing of samples is required prior to introduction into the MS instrument, and such pre-processing often results in substantial sample loss. Several sample preparation protocols designed specifically for low-input samples have been developed to combat these issues, including nanodroplet processing in one pot for trace samples (nanoPOTS) (Zhu et al., 2018; Liang et al., 2021), single-pot, solid-phase-enhanced sample preparation (SP3) (Hughes et al., 2018) and in-StageTip (iST)-based methods (Kulak et al., 2014). Proteome coverage can additionally be increased by performing pre-fractionation prior to MS analysis, which reduces the sample complexity and as such enables identification of low-abundant peptide species which would otherwise be masked by high-abundant components in an unfractionated peptide mixture (Dimayacyac-Esleta et al., 2015). In addition, improvements in liquid chromatography separation setup show promise for robust proteome profiling at high sensitivity for limited sample proteomics workflows (Stadlmann et al., 2019; Stejskal et al., 2021).

Isobaric labelling approaches have proved highly beneficial for low-input proteomics. Apart from reducing technical variability, isobaric labelling provides a means of signal amplification in MS^1^ and thus facilitates subsequent quantification and comparison across the assayed conditions. Isobaric labels have a central part in the recently developed single-cell proteomics by mass spectrometry (SCoPE-MS) technology, where protein quantification in single cells is made possible by including a larger, less refined sample in the multiplex (a so-called carrier proteome) (Budnik et al., 2018; Specht et al., 2021). This technique has been applied to resolve macrophage heterogeneity, where over 3,000 proteins across approximately 1,500 single cells were successfully quantified using the latest iteration of the method (SCoPE2) (Specht et al., 2021). Schoof and colleagues recently developed a single cell-MS workflow similar to that of SCoPE2, where they incorporated miniaturized sample preparation, isobaric labeling and state-of-the-art gas phase fractionation to perform single cell MS analysis of an AML culture model derived from primary patient samples. The optimized workflow enabled consistent quantification of roughly 1,000 proteins per cell and a throughput of 112 cells per day of instrument time (Schoof et al., 2021). While the coverage that can be achieved using the methods described by Specht et al. and Schoof et al. remains manyfold lower than what can be achieved with low-input bulk proteomics, these studies represent evidence of that the era of global single cell proteomics is upon us.

Only a handful of MS-based studies detailing the proteomic composition of primary HSPCs have been performed to date (Unwin et al., 2006; Spooncer et al., 2008; Klimmeck et al., 2012; Cabezas-Wallscheid et al., 2014; Haas et al., 2015; Jassinskaja et al., 2017, 2021; Amon et al., 2019; Zaro et al., 2020) (Table 1). The earlier of these studies utilized “classical” sample preparation methods to gain impressive coverage of the HSPC proteome from a starting material of 400,000-1,000,000 fluorescence activated cell sorting (FACS)-purified cells (Klimmeck et al., 2012; Cabezas-Wallscheid et al., 2014; Haas et al., 2015; Jassinskaja et al., 2017). In more recent work, deep proteomic coverage (>4,000 identified proteins) has been achieved in as little as 50,000 mouse and 25,000 human HSPCs by implementing iST-based sample preparation and advanced label-free MS approaches (Amon et al., 2019; Zaro et al., 2020). We recently implemented a similar iST-based approach as Zaro et al. in combination with isobaric labeling to quantify over 4,000 proteins from a starting material of 100,000 cells/sample of primary fetal and adult LMPPs, CLPs, and GMPs (Jassinskaja et al., 2021), providing further evidence for the feasibility of performing global proteomic characterization on cell types that represent a disappearingly small minority of mammalian tissues.

**TABLE 1:** Summary of studies exploring the proteomic composition of HSPCs. CMP, common myeloid progenitor; OPP, oligopotent progenitor; hu, human.

Author	Year	Cell type	Starting cell number/sample	# Protein IDs	Quantitative method
Unwin et al.	2006	HSPC, OPP	10^6^	948	DDA iTRAQ
Spooncer et al.	2008	HSPC, CMP	10^6^	1,263	DDA iTRAQ
Klimmeck et al.	2012	HSPC, CMP	10^6^	5,139	DDA Dimethyl labeling
Cabezas-Wallscheid et al.	2014	HSC, MPP1	4 × 10^5^	6,389	DDA Dimethyl labeling
Haas et al.	2015	HSC, MPP	4 × 10^5^	7,492	DDA Dimethyl labeling
Jassinskaja et al.	2017	HSPC	4.5 × 10^5^	6,909	DDA Dimethyl labeling
Amon et al.	2019	huHSPC	2.5 × 10^4^	5,851	DIA LFQ
Zaro et al.	2020	HSC, MPP, OPP	5 × 10^4^	4,030–6,035	DDA LFQ
Jassinskaja et al.	2021	LMPP, CLP, GMP	10^5^	4,189	DDA TMT

## Conclusion and Future Perspectives

State-of-the art technologies for genomic and transcriptomic characterization of cells have enabled substantial progress towards understanding the molecular mechanisms driving the differential susceptibility to leukemia in children and adults. However, a lack of knowledge about the cellular proteotype, which by many researchers is considered the best predictor of cellular behavior, has hampered improvements in treatment options and outcomes for the most aggressive subclasses of the disease. Excitingly, considering the remarkable advances made in the field of low input/single-cell proteomics over the past 15 years (Table 1), it is highly plausible that the first MS-based single-cell proteomic characterization of the hematopoietic system is only a few years away. A wider application of latest MS-based approaches for comprehensive proteome characterization of normal HSPCs, cells harboring leukemia-driving mutations, and their microenvironment, will undoubtedly aid in accelerating the development of novel targeted therapies for the treatment of aggressive blood cancers.
